# Salvaging hope: Is increasing NAD^+^ a key to treating mitochondrial myopathy?

**DOI:** 10.15252/emmm.201404179

**Published:** 2014-05-16

**Authors:** Robert N Lightowlers, Zofia MA Chrzanowska-Lightowlers

**Affiliations:** Wellcome Trust Centre for Mitochondrial Research, Institute for Cell and Molecular Biosciences, Medical School, Newcastle UniversityNewcastle upon Tyne, UK

## Abstract

Mitochondrial diseases can arise from mutations either in mitochondrial DNA or in nuclear DNA encoding mitochondrially destined proteins. Currently, there is no cure for these diseases although treatments to ameliorate a subset of the symptoms are being developed. In this issue of *EMBO Molecular Medicine*, Khan *et al* (2014) use a mouse model to test the efficacy of a simple dietary supplement of nicotinamide riboside to treat and prevent mitochondrial myopathies.

See also: **NA Khan *et al* (June 2014)**

Getting the right levels of vitamins is essential for health. Those of us of a certain age will remember in junior school being taught about the consequences of vitamin deficiency and having to memorise those consequences. For example, one deficiency, exotically named *pellagra*, resulted in a combination of dermatitis, diarrhoea and dementia. The underlying cause was identified as a lack of nicotinic acid or nicotinamide (vitamin B3). Indeed, the defect was exacerbated by a dietary lack of tryptophan. This is now understood, as all three components are important building blocks for the production of nicotinamide adenine dinucleotide, NAD, a redox-active coenzyme and enzyme substrate. This molecule is well known as a key player in metabolism, being the primary electron donor in the mitochondrial respiratory chain. It is also utilised and broken-down by a variety of proteins in other subcellular compartments, such as the family of protein deacetylases (sirtuins), the poly (ADP ribose)-phosphorylases (PARPs) and NAD glycohydrolases. *De novo* synthesis from tryptophan is a complex 8-step enzymatic process, so there are likely to be recycling pathways that utilise NAD synthesis intermediates as substrates. This is where nicotinamide and nicotinic acid feature. Both are intermediates in NAD biosynthesis, requiring enzymatic pathways of only 2 or 3 steps respectively to generate NAD (Bogan & Brenner, 2008). An additional salvage pathway has been identified in eubacteria and eukaryotes that is distinct from these nicotinic acid or nicotinamide recycling (or salvaging) pathways; in a two-step process, nicotinamide riboside (NR) can be first phosphorylated and then adenylylated to form NAD^+^ (Bieganowski & Brenner, 2004; see Fig 1). Those of us who remember memorising those vitamin deficiency diseases at school, probably also remember the compulsory bottle of milk to be drunk at break time. Although we did not realise it then, this was a good source of nicotinamide riboside, which in addition to being a normal metabolite in the body is also present in cow's milk.

**Figure 1:**
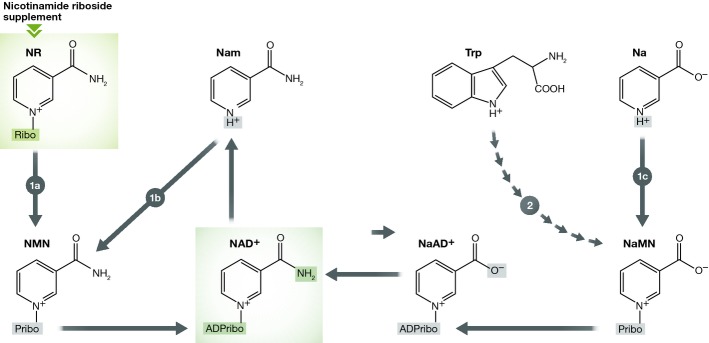
The salvage/recycling pathway for NAD^+^ biosynthesis from nicotinamide riboside (NR) in man NR, taken in to the body, can be converted to nicotinamide mononucleotide (NMN) by one of two highly conserved NR kinases in the cytoplasm (pathway 1a). NAM (nicotinamide) can also be converted by NMN synthetase to NMN (pathway 1b). NMN is further converted to NAD^+^ by the action of one of three adenylyltransferases (NMNAT1-3) that also acts on NaMN (nicotinic acid mononucleotide) to produce NaAD^+^ (nicotinic acid adenine dinucleotide). The latter is subsequently converted by NAD synthase to NAD^+^. Nicotinic acid (Na) feeds into the pathway through conversion to NaMN by Na phosphoribosyltransferase (pathway 1c). Tryptophan is the *de novo* precursor of NAD^+^ that also feeds into NaMN synthesis via a multistep pathway (2) described in Bogan and Brenner (2008).

## NR can protect against mitochondrial myopathy in mice

Defects of the mitochondrial (mt) respiratory chain constitute one of the most common forms of heritable metabolic disease. Clinical presentation varies widely, and significantly, there is no effective cure. Khan *et al* hypothesised that under conditions of respiratory chain deficiency, NADH utilisation is partially blocked leading to a decrease in the NAD^+^/NADH ratio. This constitutes a signal in the cell that is translated as indicating high nutrient availability, a condition completely at odds with the defective mitochondrial function. Therefore, by repleting levels of NAD^+^, the authors surmise that mitochondrial dysfunction could be ameliorated. To challenge their hypothesis, the authors have used their mt-Deletor mouse, a model of mitochondrial myopathy, and administered the NAD^+^ precursor, NR. The Deletor mouse carries a dominant pathogenic mutation in the major mitochondrial DNA (mtDNA) replicative helicase, Twinkle, that corresponds to a mutation found in patients (Tyynismaa *et al*, 2005). In Deletor mice, this causes increased levels of deleted mtDNA and a subtle but chronically progressive mitochondrial myopathy. Control mice and pre- and post-symptomatic Deletor mice were dosed with large (400 mg/kg/day) amounts of NR for up to 4 months, a regime previously documented to result in increased levels of NAD^+^ in skeletal muscle of wild-type mice (Canto *et al*, 2012). Khan *et al* show that this treatment resulted in a marked increase in mitochondrial biogenesis in skeletal muscle and brown adipose tissue compared to undosed controls. A similar increase had been shown in the previous experiments following NR treatment, both of cultured cells and in various mice tissue (Canto *et al*, 2012). Crucially, however, for these new NR supplement experiments, the mt-biogenesis was concomitant with a decrease in markers of disease progression in Deletor mice, which were also protected from ultrastructural abnormalities of mitochondria. NR invoked a minor increase in overall mtDNA levels in both control and Deletor mice, but intriguingly caused a decrease in the levels of deleted mtDNA that accumulated in skeletal muscle of the Deletors. Thus, data were consistent with NR treatment and increasing NAD^+^ levels protecting against mitochondrial disease in the Deletor mice. In addition to promoting mt-biogenesis, NR also appeared to enhance the mitochondrial unfolded protein response. This increase in a subset of mitochondrial chaperones and proteases is believed to be beneficial to health and promote an increased lifespan (Pellegrino *et al*, 2013).

## Why does HR treatment promote mitochondrial biogenesis?

Previous reports have implicated increased NAD^+^ levels with increased sirtuin activity, most notably SIRT1 and SIRT3 (Lagouge *et al*, 2006; Hirschey *et al*, 2011). The consequence is an activation of key transcription factors including SIRT1 and SIRT3 (Canto *et al*, 2012), which upregulate gene products that are central to mt-biogenesis (Feige *et al*, 2008). In addition to enhancing oxidative metabolism in a range of tissues, SIRT1 activation has also been reported to protect against diet-induced metabolic disorders by enhancing fatty acid oxidation (Feige *et al*, 2008). Consistent with this, Khan *et al* present data to show an NR-mediated increase in skeletal muscle mRNA levels encoding proteins that are involved in fatty acid transport or oxidation, namely CD36, ACOX1 and MCAD. Increasing mitochondrial biogenesis as a way of treating mitochondrial dysfunction is encouraging and has been previously shown to be efficacious for mouse models of mitochondrial disease (Wenz *et al*, 2008). However, it has been well described that mitochondrial proliferation can occur as a consequence of mtDNA disease in man. It will certainly be interesting to discover whether drug-induced mitochondrial biogenesis can also be beneficial to patients with mitochondrial dysfunction.

## Why are these results so encouraging?

To date, there is no effective therapy for patients with mitochondrial myopathy. Vitamin cocktails including vitamin B3 (although at far lower doses than used here) have often been used to treat such patients for many years, with only sporadic reports of efficacy. The rationale for increasing NAD^+^ levels in order to increase mitochondrial mass is reasonable, and the results reported here are compelling. What is particularly exciting is that NAD^+^ intermediates such as NR are readily available and relatively simple drugs. If the efficacy of NR is entirely due to its effects as an NAD^+^ precursor, it is not absolutely clear why neither nicotinamide nor nicotinic acid themselves could not be used. Perhaps because there is evidence that the former is hepatotoxic at high concentrations and its efficacy in increasing NAD^+^ levels in skeletal muscle is unclear (Bogan & Brenner, 2008)? Nicotinic acid, however, has been used for many years to treat patients with high serum cholesterol levels but can cause irritating vasodilation (flushing). To counter this, slow release formulations have been available for some time. Of these NAD^+^ precursors, NR or its phosphorylated NAD^+^ precursor nicotinamide mononucleotide (NMN) might be the therapeutic molecule of choice by virtue of being able to access mitochondria and be converted to NAD^+^ by mitochondrial-specific enzymes. Isoforms of the NR kinase and NMN adenylyltransferase are known, but there is conflicting evidence on their mitochondrial location (Felici *et al*, 2013). Finally, side effects following administration of other NAD^+^ precursors supplements have been reported (Sauve, 2008). It will of course be necessary to evaluate the NR dosage used by Khan *et al,* as it appears strikingly high (400 mg/kg/day) compared to most commercially available supplements (60–500 mg/person/day). Whether such a large dosage is viable as a supplement needs to be established; however, it will be exciting to follow new pharmacokinetic data for this potentially therapeutic nucleoside derivative.
